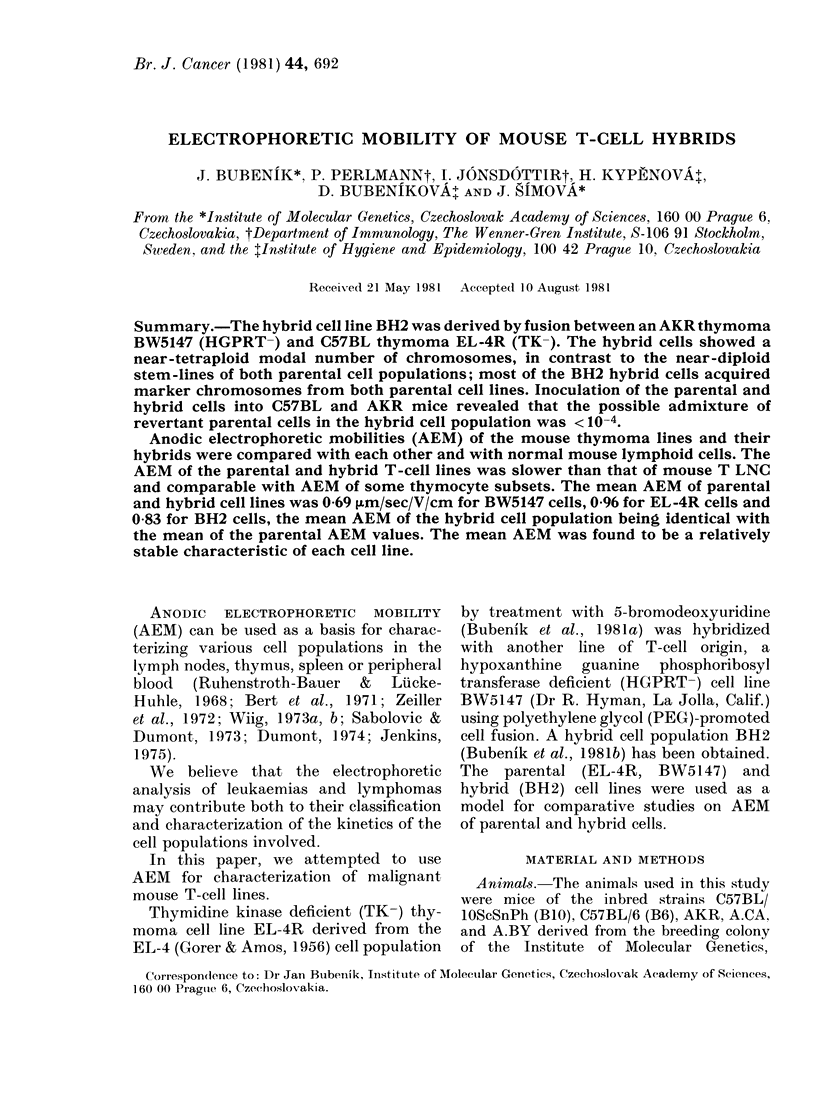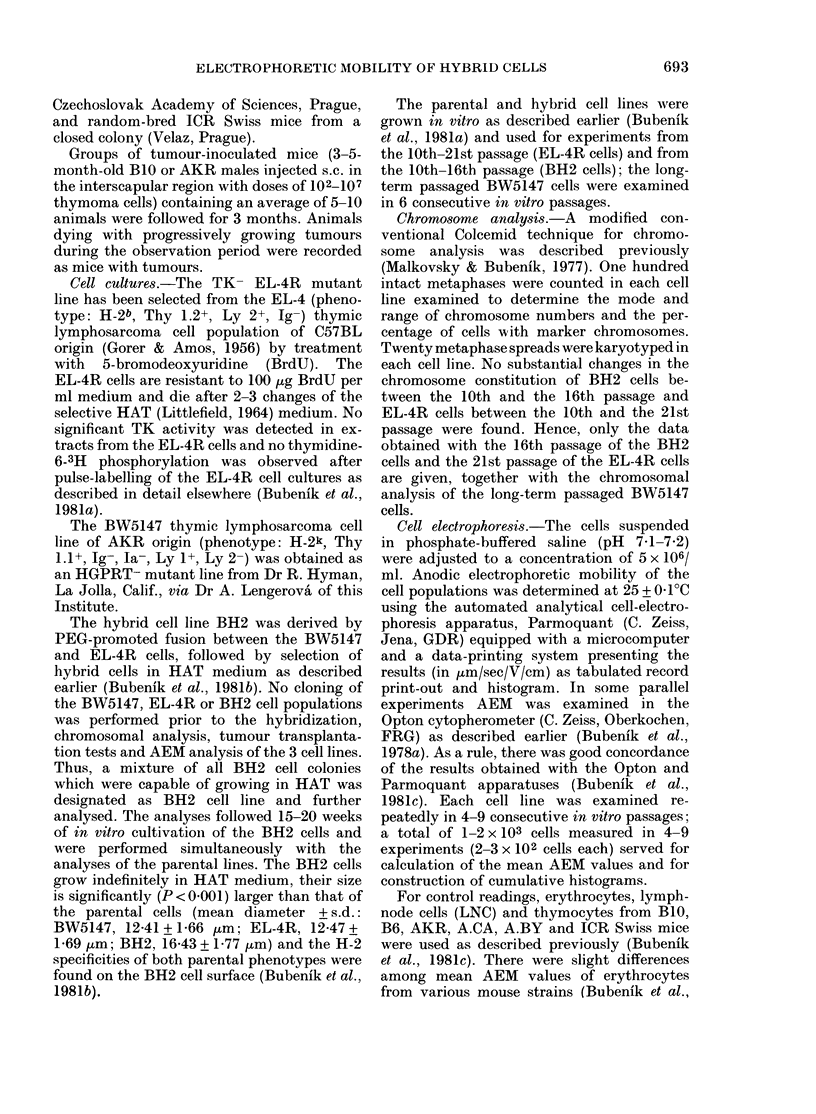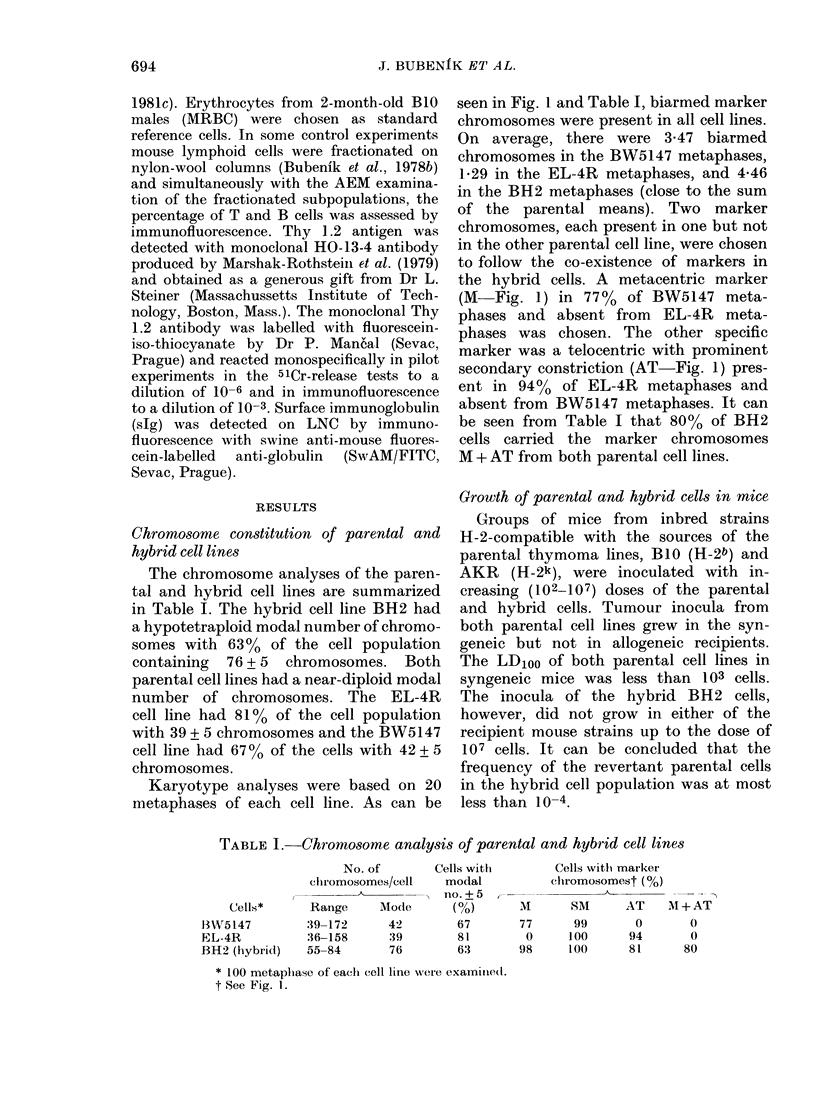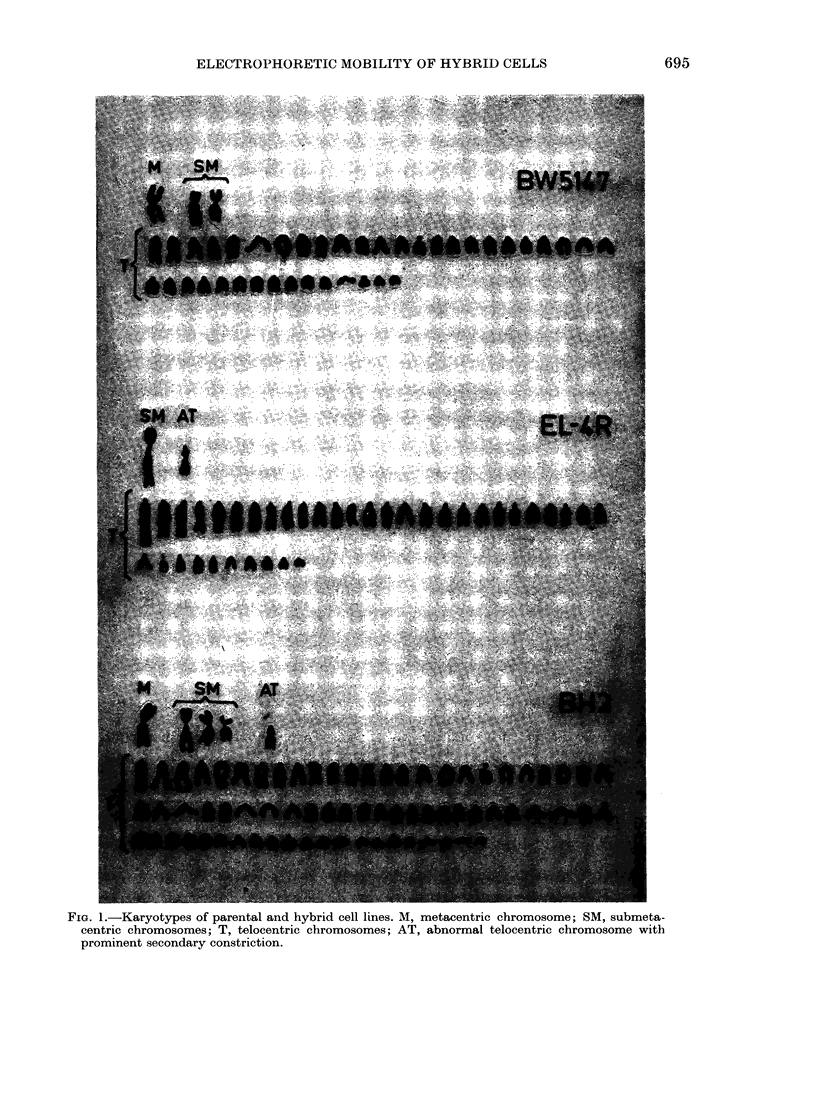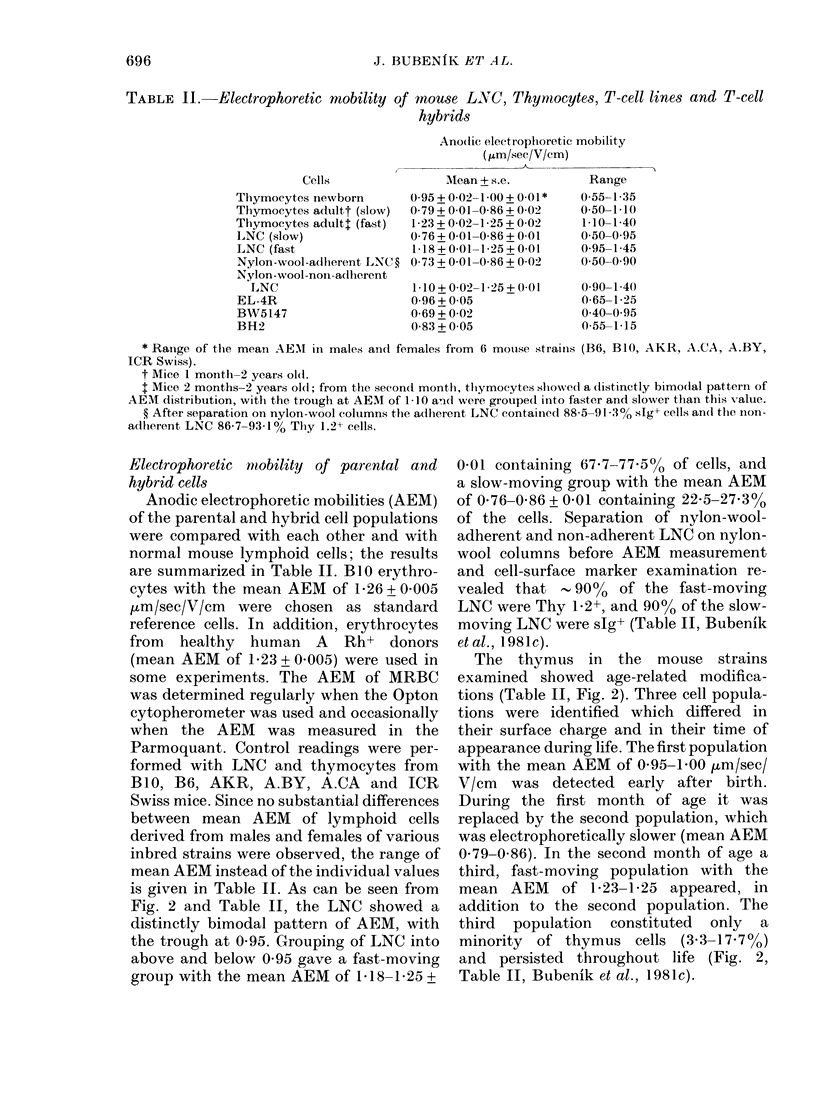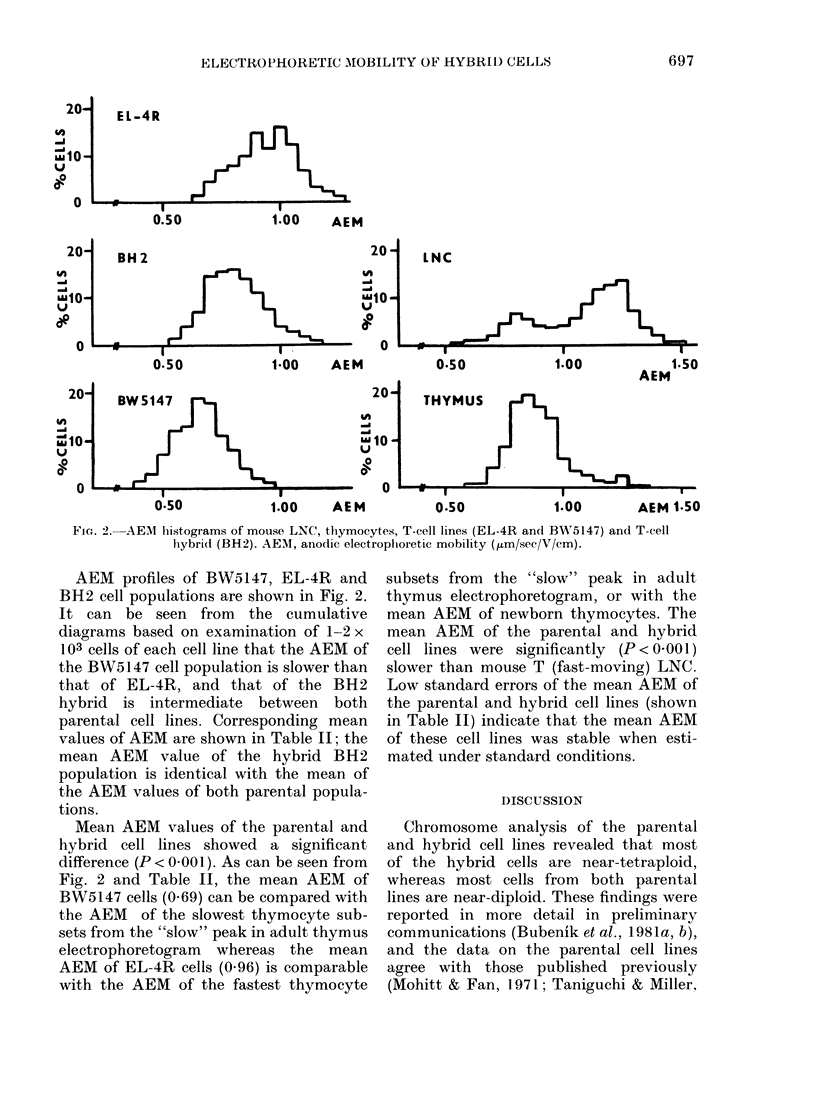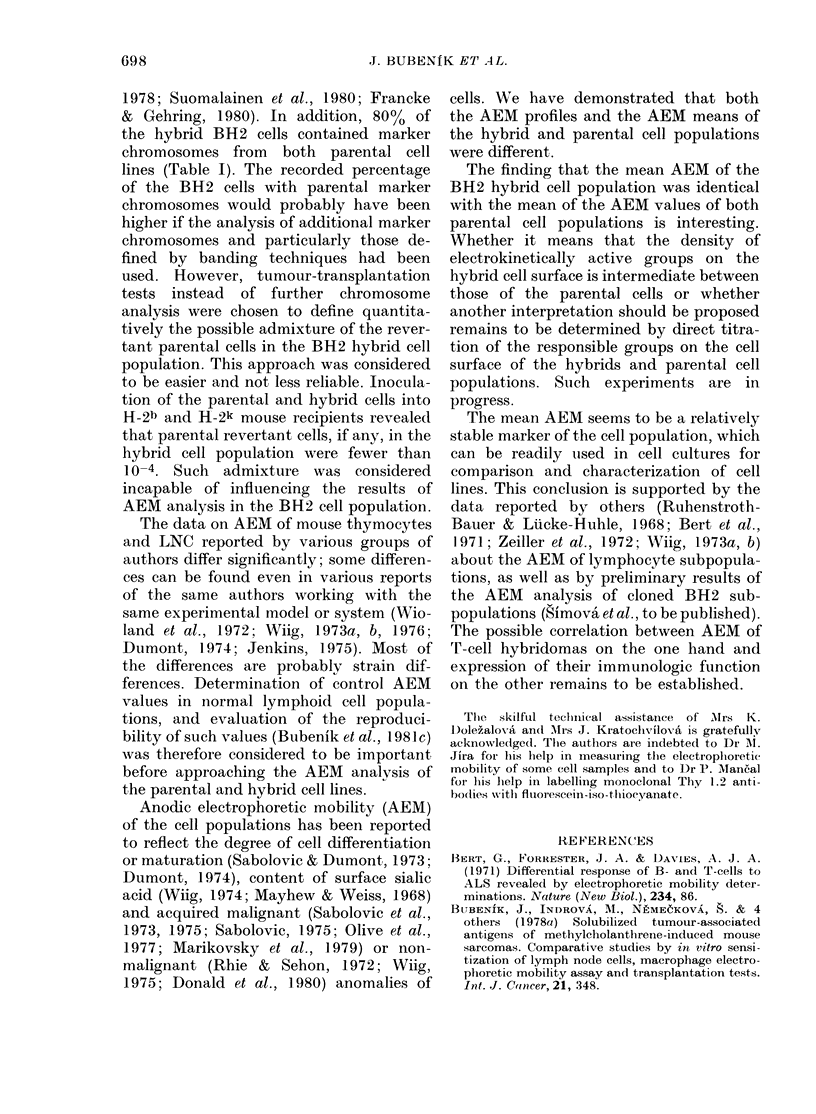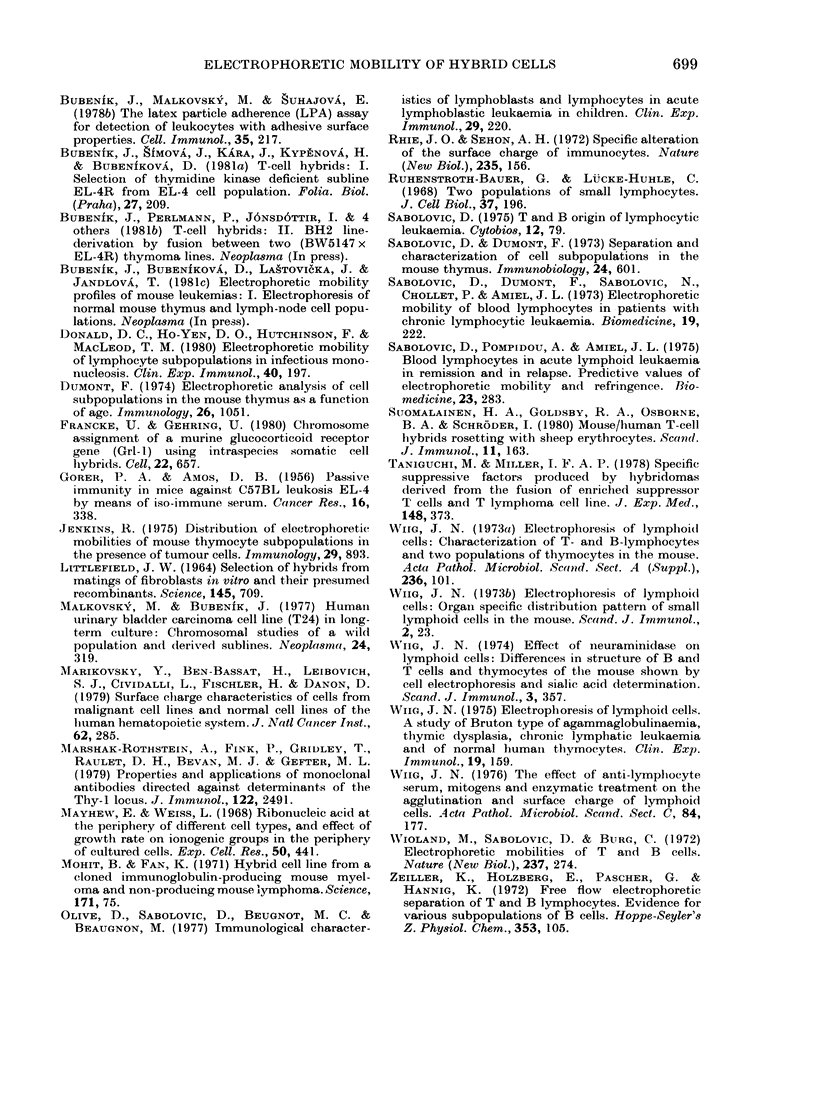# Electrophoretic mobility of mouse T-cell hybrids.

**DOI:** 10.1038/bjc.1981.255

**Published:** 1981-11

**Authors:** J. Bubeník, P. Perlmann, I. Jónsdóttir, H. Kypĕnová, D. Bubeníková, J. Símová

## Abstract

**Images:**


*Br. J. Cancer* (1981) **44**, 692

### ELECTROPHORETIC MOBILITY OF MOUSE T-CELL HYBRIDS

J. BUBENÍK*, P. PERLMANN†, I. JÓNSDÓTTIR†, H. KYPĚNOVÁ‡, D. BUBENÍKOVÁ‡ AND J. ŠÍMOVÁ*

*From the *Institute of Molecular Genetics, Czechoslovak Academy of Sciences,* 160 00 *Prague* 6, *Czechoslovakia,* †*Department of Immunology, The Wenner-Gren Institute, S*-106 91 *Stockholm, Sweden, and the* ‡*Institute of Hygiene and Epidemiology,* 100 42 *Prague* 10, *Czechoslovakia*

Received 21 May 1981 Accepted 10 August 1981

**Summary.—The hybrid cell line BH2 was derived by fusion between an AKR thymoma BW5147 (HGPRT⁻) and C57BL thymoma EL-4R (TK⁻). The hybrid cells showed a near-tetraploid modal number of chromosomes, in contrast to the near-diploid stem-lines of both parental cell populations; most of the BH2 hybrid cells acquired marker chromosomes from both parental cell lines. Inoculation of the parental and hybrid cells into C57BL and AKR mice revealed that the possible admixture of revertant parental cells in the hybrid cell population was $<10^{-4}$.**

**Anodic electrophoretic mobilities (AEM) of the mouse thymoma lines and their hybrids were compared with each other and with normal mouse lymphoid cells. The AEM of the parental and hybrid T-cell lines was slower than that of mouse T LNC and comparable with AEM of some thymocyte subsets. The mean AEM of parental and hybrid cell lines was 0·69 μm/sec/V/cm for BW5147 cells, 0·96 for EL-4R cells and 0·83 for BH2 cells, the mean AEM of the hybrid cell population being identical with the mean of the parental AEM values. The mean AEM was found to be a relatively stable characteristic of each cell line.**

ANODIC ELECTROPHORETIC MOBILITY (AEM) can be used as a basis for characterizing various cell populations in the lymph nodes, thymus, spleen or peripheral blood (Ruhenstroth-Bauer & Lücke-Huhle, 1968; Bert *et al.*, 1971; Zeiller *et al.*, 1972; Wiig, 1973*a*, *b*; Sabolovic & Dumont, 1973; Dumont, 1974; Jenkins, 1975).

We believe that the electrophoretic analysis of leukaemias and lymphomas may contribute both to their classification and characterization of the kinetics of the cell populations involved.

In this paper, we attempted to use AEM for characterization of malignant mouse T-cell lines.

Thymidine kinase deficient ($TK^-$) thymoma cell line EL-4R derived from the EL-4 (Gorer & Amos, 1956) cell population by treatment with 5-bromodeoxyuridine (Bubeník *et al.*, 1981*a*) was hybridized with another line of T-cell origin, a hypoxanthine guanine phosphoribosyl transferase deficient ($HGPRT^-$) cell line BW5147 (Dr R. Hyman, La Jolla, Calif.) using polyethylene glycol (PEG)-promoted cell fusion. A hybrid cell population BH2 (Bubeník *et al.*, 1981*b*) has been obtained. The parental (EL-4R, BW5147) and hybrid (BH2) cell lines were used as a model for comparative studies on AEM of parental and hybrid cells.

### MATERIAL AND METHODS

*Animals.*—The animals used in this study were mice of the inbred strains C57BL/10ScSnPh (B10), C57BL/6 (B6), AKR, A.CA, and A.BY derived from the breeding colony of the Institute of Molecular Genetics,

Correspondence to: Dr Jan Bubeník, Institute of Molecular Genetics, Czechoslovak Academy of Sciences, 160 00 Prague 6, Czechoslovakia.

Czechoslovak Academy of Sciences, Prague, and random-bred ICR Swiss mice from a closed colony (Velaz, Prague).

Groups of tumour-inoculated mice (3–5-month-old B10 or AKR males injected s.c. in the interscapular region with doses of $10^2$–$10^7$ thymoma cells) containing an average of 5–10 animals were followed for 3 months. Animals dying with progressively growing tumours during the observation period were recorded as mice with tumours.

*Cell cultures.*—The $TK^-$ EL-4R mutant line has been selected from the EL-4 (phenotype: H-$2^b$, Thy $1.2^+$, Ly $2^+$, $Ig^-$) thymic lymphosarcoma cell population of C57BL origin (Gorer & Amos, 1956) by treatment with 5-bromodeoxyuridine (BrdU). The EL-4R cells are resistant to 100 $\mu$g BrdU per ml medium and die after 2–3 changes of the selective HAT (Littlefield, 1964) medium. No significant TK activity was detected in extracts from the EL-4R cells and no thymidine-6-$^3$H phosphorylation was observed after pulse-labelling of the EL-4R cell cultures as described in detail elsewhere (Bubeník *et al.*, 1981*a*).

The BW5147 thymic lymphosarcoma cell line of AKR origin (phenotype: H-$2^k$, Thy $1.1^+$, $Ig^-$, $Ia^-$, Ly $1^+$, Ly $2^-$) was obtained as an $HGPRT^-$ mutant line from Dr R. Hyman, La Jolla, Calif., *via* Dr A. Lengerová of this Institute.

The hybrid cell line BH2 was derived by PEG-promoted fusion between the BW5147 and EL-4R cells, followed by selection of hybrid cells in HAT medium as described earlier (Bubeník *et al.*, 1981*b*). No cloning of the BW5147, EL-4R or BH2 cell populations was performed prior to the hybridization, chromosomal analysis, tumour transplantation tests and AEM analysis of the 3 cell lines. Thus, a mixture of all BH2 cell colonies which were capable of growing in HAT was designated as BH2 cell line and further analysed. The analyses followed 15–20 weeks of *in vitro* cultivation of the BH2 cells and were performed simultaneously with the analyses of the parental lines. The BH2 cells grow indefinitely in HAT medium, their size is significantly ($P < 0{\cdot}001$) larger than that of the parental cells (mean diameter $\pm$s.d.: BW5147, $12{\cdot}41 \pm 1{\cdot}66$ $\mu$m; EL-4R, $12{\cdot}47 \pm 1{\cdot}69$ $\mu$m; BH2, $16{\cdot}43 \pm 1{\cdot}77$ $\mu$m) and the H-2 specificities of both parental phenotypes were found on the BH2 cell surface (Bubeník *et al.*, 1981*b*).

The parental and hybrid cell lines were grown *in vitro* as described earlier (Bubeník *et al.*, 1981*a*) and used for experiments from the 10th–21st passage (EL-4R cells) and from the 10th–16th passage (BH2 cells); the long-term passaged BW5147 cells were examined in 6 consecutive *in vitro* passages.

*Chromosome analysis.*—A modified conventional Colcemid technique for chromosome analysis was described previously (Malkovsky & Bubeník, 1977). One hundred intact metaphases were counted in each cell line examined to determine the mode and range of chromosome numbers and the percentage of cells with marker chromosomes. Twenty metaphase spreads were karyotyped in each cell line. No substantial changes in the chromosome constitution of BH2 cells between the 10th and the 16th passage and EL-4R cells between the 10th and the 21st passage were found. Hence, only the data obtained with the 16th passage of the BH2 cells and the 21st passage of the EL-4R cells are given, together with the chromosomal analysis of the long-term passaged BW5147 cells.

*Cell electrophoresis.*—The cells suspended in phosphate-buffered saline (pH 7·1–7·2) were adjusted to a concentration of $5 \times 10^6$/ml. Anodic electrophoretic mobility of the cell populations was determined at $25 \pm 0{\cdot}1$°C using the automated analytical cell-electrophoresis apparatus, Parmoquant (C. Zeiss, Jena, GDR) equipped with a microcomputer and a data-printing system presenting the results (in $\mu$m/sec/V/cm) as tabulated record print-out and histogram. In some parallel experiments AEM was examined in the Opton cytopherometer (C. Zeiss, Oberkochen, FRG) as described earlier (Bubeník *et al.*, 1978*a*). As a rule, there was good concordance of the results obtained with the Opton and Parmoquant apparatuses (Bubeník *et al.*, 1981*c*). Each cell line was examined repeatedly in 4–9 consecutive *in vitro* passages; a total of $1$–$2 \times 10^3$ cells measured in 4–9 experiments ($2$–$3 \times 10^2$ cells each) served for calculation of the mean AEM values and for construction of cumulative histograms.

For control readings, erythrocytes, lymph-node cells (LNC) and thymocytes from B10, B6, AKR, A.CA, A.BY and ICR Swiss mice were used as described previously (Bubeník *et al.*, 1981*c*). There were slight differences among mean AEM values of erythrocytes from various mouse strains (Bubeník *et al.*,

1981*c*). Erythrocytes from 2-month-old B10 males (MRBC) were chosen as standard reference cells. In some control experiments mouse lymphoid cells were fractionated on nylon-wool columns (Bubeník *et al.*, 1978*b*) and simultaneously with the AEM examination of the fractionated subpopulations, the percentage of T and B cells was assessed by immunofluorescence. Thy 1.2 antigen was detected with monoclonal HO-13-4 antibody produced by Marshak-Rothstein *et al.* (1979) and obtained as a generous gift from Dr L. Steiner (Massachussetts Institute of Technology, Boston, Mass.). The monoclonal Thy 1.2 antibody was labelled with fluorescein-iso-thiocyanate by Dr P. Mančal (Sevac, Prague) and reacted monospecifically in pilot experiments in the $^{51}Cr$-release tests to a dilution of $10^{-6}$ and in immunofluorescence to a dilution of $10^{-3}$. Surface immunoglobulin (sIg) was detected on LNC by immunofluorescence with swine anti-mouse fluorescein-labelled anti-globulin (SwAM/FITC, Sevac, Prague).

### RESULTS

### *Chromosome constitution of parental and hybrid cell lines*

The chromosome analyses of the parental and hybrid cell lines are summarized in Table I. The hybrid cell line BH2 had a hypotetraploid modal number of chromosomes with 63% of the cell population containing $76 \pm 5$ chromosomes. Both parental cell lines had a near-diploid modal number of chromosomes. The EL-4R cell line had 81% of the cell population with $39 \pm 5$ chromosomes and the BW5147 cell line had 67% of the cells with $42 \pm 5$ chromosomes.

Karyotype analyses were based on 20 metaphases of each cell line. As can be seen in Fig. 1 and Table I, biarmed marker chromosomes were present in all cell lines. On average, there were 3·47 biarmed chromosomes in the BW5147 metaphases, 1·29 in the EL-4R metaphases, and 4·46 in the BH2 metaphases (close to the sum of the parental means). Two marker chromosomes, each present in one but not in the other parental cell line, were chosen to follow the co-existence of markers in the hybrid cells. A metacentric marker (M—Fig. 1) in 77% of BW5147 metaphases and absent from EL-4R metaphases was chosen. The other specific marker was a telocentric with prominent secondary constriction (AT—Fig. 1) present in 94% of EL-4R metaphases and absent from BW5147 metaphases. It can be seen from Table I that 80% of BH2 cells carried the marker chromosomes M + AT from both parental cell lines.

### *Growth of parental and hybrid cells in mice*

Groups of mice from inbred strains H-2-compatible with the sources of the parental thymoma lines, B10 (H-$2^b$) and AKR (H-$2^k$), were inoculated with increasing ($10^2$–$10^7$) doses of the parental and hybrid cells. Tumour inocula from both parental cell lines grew in the syngeneic but not in allogeneic recipients. The $LD_{100}$ of both parental cell lines in syngeneic mice was less than $10^3$ cells. The inocula of the hybrid BH2 cells, however, did not grow in either of the recipient mouse strains up to the dose of $10^7$ cells. It can be concluded that the frequency of the revertant parental cells in the hybrid cell population was at most less than $10^{-4}$.

TABLE I.—*Chromosome analysis of parental and hybrid cell lines*

| Cells* | No. of chromosomes/cell: Range | Mode | Cells with modal no. ± 5 (%) | Cells with marker chromosomes† (%): M | SM | AT | M + AT |
|---|---|---|---|---|---|---|---|
| BW5147 | 39–172 | 42 | 67 | 77 | 99 | 0 | 0 |
| EL-4R | 36–158 | 39 | 81 | 0 | 100 | 94 | 0 |
| BH2 (hybrid) | 55–84 | 76 | 63 | 98 | 100 | 81 | 80 |

* 100 metaphase of each cell line were examined.

† See Fig. 1.

FIG. 1.—Karyotypes of parental and hybrid cell lines. M, metacentric chromosome; SM, submetacentric chromosomes; T, telocentric chromosomes; AT, abnormal telocentric chromosome with prominent secondary constriction.

TABLE II.—*Electrophoretic mobility of mouse LNC, Thymocytes, T-cell lines and T-cell hybrids*

| Cells | Anodic electrophoretic mobility (μm/sec/V/cm) Mean ± s.e. | Range |
|---|---|---|
| Thymocytes newborn | 0·95 ± 0·02–1·00 ± 0·01* | 0·55–1·35 |
| Thymocytes adult† (slow) | 0·79 ± 0·01–0·86 ± 0·02 | 0·50–1·10 |
| Thymocytes adult‡ (fast) | 1·23 ± 0·02–1·25 ± 0·02 | 1·10–1·40 |
| LNC (slow) | 0·76 ± 0·01–0·86 ± 0·01 | 0·50–0·95 |
| LNC (fast | 1·18 ± 0·01–1·25 ± 0·01 | 0·95–1·45 |
| Nylon-wool-adherent LNC§ | 0·73 ± 0·01–0·86 ± 0·02 | 0·50–0·90 |
| Nylon-wool-non-adherent LNC | 1·10 ± 0·02–1·25 ± 0·01 | 0·90–1·40 |
| EL-4R | 0·96 ± 0·05 | 0·65–1·25 |
| BW5147 | 0·69 ± 0·02 | 0·40–0·95 |
| BH2 | 0·83 ± 0·05 | 0·55–1·15 |

* Range of the mean AEM in males and females from 6 mouse strains (B6, B10, AKR, A.CA, A.BY, ICR Swiss).

† Mice 1 month–2 years old.

‡ Mice 2 months–2 years old; from the second month, thymocytes showed a distinctly bimodal pattern of AEM distribution, with the trough at AEM of 1·10 and were grouped into faster and slower than this value.

§ After separation on nylon-wool columns the adherent LNC contained 88·5–91·3% sIg$^+$ cells and the non-adherent LNC 86·7–93·1% Thy 1.2$^+$ cells.

*Electrophoretic mobility of parental and hybrid cells*

Anodic electrophoretic mobilities (AEM) of the parental and hybrid cell populations were compared with each other and with normal mouse lymphoid cells; the results are summarized in Table II. B10 erythrocytes with the mean AEM of 1·26 ± 0·005 μm/sec/V/cm were chosen as standard reference cells. In addition, erythrocytes from healthy human A Rh$^+$ donors (mean AEM of 1·23 ± 0·005) were used in some experiments. The AEM of MRBC was determined regularly when the Opton cytopherometer was used and occasionally when the AEM was measured in the Parmoquant. Control readings were performed with LNC and thymocytes from B10, B6, AKR, A.BY, A.CA and ICR Swiss mice. Since no substantial differences between mean AEM of lymphoid cells derived from males and females of various inbred strains were observed, the range of mean AEM instead of the individual values is given in Table II. As can be seen from Fig. 2 and Table II, the LNC showed a distinctly bimodal pattern of AEM, with the trough at 0·95. Grouping of LNC into above and below 0·95 gave a fast-moving group with the mean AEM of 1·18–1·25 ± 0·01 containing 67·7–77·5% of cells, and a slow-moving group with the mean AEM of 0·76–0·86 ± 0·01 containing 22·5–27·3% of the cells. Separation of nylon-wool-adherent and non-adherent LNC on nylon-wool columns before AEM measurement and cell-surface marker examination revealed that ~90% of the fast-moving LNC were Thy 1·2$^+$, and 90% of the slow-moving LNC were sIg$^+$ (Table II, Bubeník *et al.*, 1981*c*).

The thymus in the mouse strains examined showed age-related modifications (Table II, Fig. 2). Three cell populations were identified which differed in their surface charge and in their time of appearance during life. The first population with the mean AEM of 0·95–1·00 μm/sec/V/cm was detected early after birth. During the first month of age it was replaced by the second population, which was electrophoretically slower (mean AEM 0·79–0·86). In the second month of age a third, fast-moving population with the mean AEM of 1·23–1·25 appeared, in addition to the second population. The third population constituted only a minority of thymus cells (3·3–17·7%) and persisted throughout life (Fig. 2, Table II, Bubeník *et al.*, 1981*c*).

FIG. 2.—AEM histograms of mouse LNC, thymocytes, T-cell lines (EL-4R and BW5147) and T-cell hybrid (BH2). AEM, anodic electrophoretic mobility (μm/sec/V/cm).

AEM profiles of BW5147, EL-4R and BH2 cell populations are shown in Fig. 2. It can be seen from the cumulative diagrams based on examination of 1–2 × $10^3$ cells of each cell line that the AEM of the BW5147 cell population is slower than that of EL-4R, and that of the BH2 hybrid is intermediate between both parental cell lines. Corresponding mean values of AEM are shown in Table II; the mean AEM value of the hybrid BH2 population is identical with the mean of the AEM values of both parental populations.

Mean AEM values of the parental and hybrid cell lines showed a significant difference ($P < 0{\cdot}001$). As can be seen from Fig. 2 and Table II, the mean AEM of BW5147 cells (0·69) can be compared with the AEM of the slowest thymocyte subsets from the "slow" peak in adult thymus electrophoretogram whereas the mean AEM of EL-4R cells (0·96) is comparable with the AEM of the fastest thymocyte subsets from the "slow" peak in adult thymus electrophoretogram, or with the mean AEM of newborn thymocytes. The mean AEM of the parental and hybrid cell lines were significantly ($P < 0{\cdot}001$) slower than mouse T (fast-moving) LNC. Low standard errors of the mean AEM of the parental and hybrid cell lines (shown in Table II) indicate that the mean AEM of these cell lines was stable when estimated under standard conditions.

### DISCUSSION

Chromosome analysis of the parental and hybrid cell lines revealed that most of the hybrid cells are near-tetraploid, whereas most cells from both parental lines are near-diploid. These findings were reported in more detail in preliminary communications (Bubeník *et al.*, 1981*a*, *b*), and the data on the parental cell lines agree with those published previously (Mohitt & Fan, 1971; Taniguchi & Miller,

1978; Suomalainen *et al.*, 1980; Francke & Gehring, 1980). In addition, 80% of the hybrid BH2 cells contained marker chromosomes from both parental cell lines (Table I). The recorded percentage of the BH2 cells with parental marker chromosomes would probably have been higher if the analysis of additional marker chromosomes and particularly those defined by banding techniques had been used. However, tumour-transplantation tests instead of further chromosome analysis were chosen to define quantitatively the possible admixture of the revertant parental cells in the BH2 hybrid cell population. This approach was considered to be easier and not less reliable. Inoculation of the parental and hybrid cells into $H\text{-}2^b$ and $H\text{-}2^k$ mouse recipients revealed that parental revertant cells, if any, in the hybrid cell population were fewer than $10^{-4}$. Such admixture was considered incapable of influencing the results of AEM analysis in the BH2 cell population.

The data on AEM of mouse thymocytes and LNC reported by various groups of authors differ significantly; some differences can be found even in various reports of the same authors working with the same experimental model or system (Wioland *et al.*, 1972; Wiig, 1973*a*, *b*, 1976; Dumont, 1974; Jenkins, 1975). Most of the differences are probably strain differences. Determination of control AEM values in normal lymphoid cell populations, and evaluation of the reproducibility of such values (Bubeník *et al.*, 1981*c*) was therefore considered to be important before approaching the AEM analysis of the parental and hybrid cell lines.

Anodic electrophoretic mobility (AEM) of the cell populations has been reported to reflect the degree of cell differentiation or maturation (Sabolovic & Dumont, 1973; Dumont, 1974), content of surface sialic acid (Wiig, 1974; Mayhew & Weiss, 1968) and acquired malignant (Sabolovic *et al.*, 1973, 1975; Sabolovic, 1975; Olive *et al.*, 1977; Marikovsky *et al.*, 1979) or nonmalignant (Rhie & Sehon, 1972; Wiig, 1975; Donald *et al.*, 1980) anomalies of cells. We have demonstrated that both the AEM profiles and the AEM means of the hybrid and parental cell populations were different.

The finding that the mean AEM of the BH2 hybrid cell population was identical with the mean of the AEM values of both parental cell populations is interesting. Whether it means that the density of electrokinetically active groups on the hybrid cell surface is intermediate between those of the parental cells or whether another interpretation should be proposed remains to be determined by direct titration of the responsible groups on the cell surface of the hybrids and parental cell populations. Such experiments are in progress.

The mean AEM seems to be a relatively stable marker of the cell population, which can be readily used in cell cultures for comparison and characterization of cell lines. This conclusion is supported by the data reported by others (Ruhenstroth-Bauer & Lücke-Huhle, 1968; Bert *et al.*, 1971; Zeiller *et al.*, 1972; Wiig, 1973*a*, *b*) about the AEM of lymphocyte subpopulations, as well as by preliminary results of the AEM analysis of cloned BH2 subpopulations (Šímová *et al.*, to be published). The possible correlation between AEM of T-cell hybridomas on the one hand and expression of their immunologic function on the other remains to be established.

The skilful technical assistance of Mrs K. Doležalová and Mrs J. Kratochvílová is gratefully acknowledged. The authors are indebted to Dr M. Jíra for his help in measuring the electrophoretic mobility of some cell samples and to Dr P. Mančal for his help in labelling monoclonal Thy 1.2 antibodies with fluorescein-iso-thiocyanate.

### REFERENCES

BERT, G., FORRESTER, J. A. & DAVIES, A. J. A. (1971) Differential response of B- and T-cells to ALS revealed by electrophoretic mobility determinations. *Nature* (*New Biol.*), **234**, 86.

BUBENÍK, J., INDROVÁ, M., NĚMEČKOVÁ, Š. & 4 others (1978*a*) Solubilized tumour-associated antigens of methylcholanthrene-induced mouse sarcomas. Comparative studies by *in vitro* sensitization of lymph node cells, macrophage electrophoretic mobility assay and transplantation tests. *Int. J. Cancer*, **21**, 348.

BUBENÍK, J., MALKOVSKÝ, M. & ŠUHAJOVÁ, E. (1978*b*) The latex particle adherence (LPA) assay for detection of leukocytes with adhesive surface properties. *Cell. Immunol.*, **35**, 217.

BUBENÍK, J., ŠÍMOVÁ, J., KÁRA, J., KYPĚNOVÁ, H. & BUBENÍKOVÁ, D. (1981*a*) T-cell hybrids: I. Selection of thymidine kinase deficient subline EL-4R from EL-4 cell population. *Folia. Biol.* (*Praha*), **27**, 209.

BUBENÍK, J., PERLMANN, P., JÓNSDÓTTIR, I. & 4 others (1981*b*) T-cell hybrids: II. BH2 line-derivation by fusion between two (BW5147 × EL-4R) thymoma lines. *Neoplasma* (In press).

BUBENÍK, J., BUBENÍKOVÁ, D., LAŠTOVIČKA, J. & JANDLOVÁ, T. (1981*c*) Electrophoretic mobility profiles of mouse leukemias: I. Electrophoresis of normal mouse thymus and lymph-node cell populations. *Neoplasma* (In press).

DONALD, D. C., HO-YEN, D. O., HUTCHINSON, F. & MACLEOD, T. M. (1980) Electrophoretic mobility of lymphocyte subpopulations in infectious mononucleosis. *Clin. Exp. Immunol.*, **40**, 197.

DUMONT, F. (1974) Electrophoretic analysis of cell subpopulations in the mouse thymus as a function of age. *Immunology*, **26**, 1051.

FRANCKE, U. & GEHRING, U. (1980) Chromosome assignment of a murine glucocorticoid receptor gene (Grl-1) using intraspecies somatic cell hybrids. *Cell*, **22**, 657.

GORER, P. A. & AMOS, D. B. (1956) Passive immunity in mice against C57BL leukosis EL-4 by means of iso-immune serum. *Cancer Res.*, **16**, 338.

JENKINS, R. (1975) Distribution of electrophoretic mobilities of mouse thymocyte subpopulations in the presence of tumour cells. *Immunology*, **29**, 893.

LITTLEFIELD, J. W. (1964) Selection of hybrids from matings of fibroblasts *in vitro* and their presumed recombinants. *Science*, **145**, 709.

MALKOVSKÝ, M. & BUBENÍK, J. (1977) Human urinary bladder carcinoma cell line (T24) in long-term culture: Chromosomal studies of a wild population and derived sublines. *Neoplasma*, **24**, 319.

MARIKOVSKY, Y., BEN-BASSAT, H., LEIBOVICH, S. J., CIVIDALLI, L., FISCHLER, H. & DANON, D. (1979) Surface charge characteristics of cells from malignant cell lines and normal cell lines of the human hematopoietic system. *J. Natl Cancer Inst.*, **62**, 285.

MARSHAK-ROTHSTEIN, A., FINK, P., GRIDLEY, T., RAULET, D. H., BEVAN, M. J. & GEFTER, M. L. (1979) Properties and applications of monoclonal antibodies directed against determinants of the Thy-1 locus. *J. Immunol.*, **122**, 2491.

MAYHEW, E. & WEISS, L. (1968) Ribonucleic acid at the periphery of different cell types, and effect of growth rate on ionogenic groups in the periphery of cultured cells. *Exp. Cell. Res.*, **50**, 441.

MOHIT, B. & FAN, K. (1971) Hybrid cell line from a cloned immunoglobulin-producing mouse myeloma and non-producing mouse lymphoma. *Science*, **171**, 75.

OLIVE, D., SABOLOVIC, D., BEUGNOT, M. C. & BEAUGNON, M. (1977) Immunological characteristics of lymphoblasts and lymphocytes in acute lymphoblastic leukaemia in children. *Clin. Exp. Immunol.*, **29**, 220.

RHIE, J. O. & SEHON, A. H. (1972) Specific alteration of the surface charge of immunocytes. *Nature* (*New Biol.*), **235**, 156.

RUHENSTROTH-BAUER, G. & LÜCKE-HUHLE, C. (1968) Two populations of small lymphocytes. *J. Cell Biol.*, **37**, 196.

SABOLOVIC, D. (1975) T and B origin of lymphocytic leukaemia. *Cytobios*, **12**, 79.

SABOLOVIC, D. & DUMONT, F. (1973) Separation and characterization of cell subpopulations in the mouse thymus. *Immunobiology*, **24**, 601.

SABOLOVIC, D., DUMONT, F., SABOLOVIC, N., CHOLLET, P. & AMIEL, J. L. (1973) Electrophoretic mobility of blood lymphocytes in patients with chronic lymphocytic leukaemia. *Biomedicine*, **19**, 222.

SABOLOVIC, D., POMPIDOU, A. & AMIEL, J. L. (1975) Blood lymphocytes in acute lymphoid leukaemia in remission and in relapse. Predictive values of electrophoretic mobility and refringence. *Biomedicine*, **23**, 283.

SUOMALAINEN, H. A., GOLDSBY, R. A., OSBORNE, B. A. & SCHRÖDER, I. (1980) Mouse/human T-cell hybrids rosetting with sheep erythrocytes. *Scand. J. Immunol.*, **11**, 163.

TANIGUCHI, M. & MILLER, I. F. A. P. (1978) Specific suppressive factors produced by hybridomas derived from the fusion of enriched suppressor T cells and T lymphoma cell line. *J. Exp. Med.*, **148**, 373.

WIIG, J. N. (1973*a*) Electrophoresis of lymphoid cells: Characterization of T- and B-lymphocytes and two populations of thymocytes in the mouse. *Acta Pathol. Microbiol. Scand. Sect. A* (*Suppl.*), **236**, 101.

WIIG, J. N. (1973*b*) Electrophoresis of lymphoid cells: Organ specific distribution pattern of small lymphoid cells in the mouse. *Scand. J. Immunol.*, **2**, 23.

WIIG, J. N. (1974) Effect of neuraminidase on lymphoid cells: Differences in structure of B and T cells and thymocytes of the mouse shown by cell electrophoresis and sialic acid determination. *Scand. J. Immunol.*, **3**, 357.

WIIG, J. N. (1975) Electrophoresis of lymphoid cells. A study of Bruton type of agammaglobulinaemia, thymic dysplasia, chronic lymphatic leukaemia and of normal human thymocytes. *Clin. Exp. Immunol.*, **19**, 159.

WIIG, J. N. (1976) The effect of anti-lymphocyte serum, mitogens and enzymatic treatment on the agglutination and surface charge of lymphoid cells. *Acta Pathol. Microbiol. Scand. Sect. C*, **84**, 177.

WIOLAND, M., SABOLOVIC, D. & BURG, C. (1972) Electrophoretic mobilities of T and B cells. *Nature* (*New Biol.*), **237**, 274.

ZEILLER, K., HOLZBERG, E., PASCHER, G. & HANNIG, K. (1972) Free flow electrophoretic separation of T and B lymphocytes. Evidence for various subpopulations of B cells. *Hoppe-Seyler's Z. Physiol. Chem.*, **353**, 105.